# Knockdown of lncRNA ACTA2-AS1 reverses cisplatin resistance of ovarian cancer cells via inhibition of miR-378a-3p-regulated Wnt5a

**DOI:** 10.1080/21655979.2022.2061181

**Published:** 2022-04-12

**Authors:** Chenxiao Lin, Meiyun Zheng, Youlin Yang, Yi Chen, Xiahui Zhang, Lingping Zhu, Haiyan Zhang

**Affiliations:** Department of Obstetrics and Gynecology, The First People’s Hospital of Wenling, Wenling, Zhejiang, China

**Keywords:** DDP resistance, ovarian cancer, ACTA2-AS1, miR-378a-3p, Wnt5a

## Abstract

Cisplatin (DDP) resistance is a principal cause leading to poor prognosis in females suffering from ovarian cancer (OC). Long non-coding RNA (lncRNA) has been shown to have an involvement in regulating cellular processes; chemoresistance being one of them the precise object of this work was to probe into the role of lncRNA ACTA2-AS1 in OC cells that have developed DDP resistance. We developed DDP-resistant OC cell lines (A2780/DDP and SKOV3/DDP). The influence of the ACTA2-AS1/miR-378a-3p/Wnt5a axis on DDP chemoresistance of DDP-resistant OC cells was ascertained using real-time PCR, Elisa, and CCK-8, and dual-luciferase reporter assay. In DDP-resistant cells and tissues, ACTA2-AS1 was increased, while a substantial downregulation in miR-378a-3p was noticed. In cells manifesting DDP-resistance, knocking down ACTA2-AS1 boosted the expression of miR-378a-3p. Further research into the mechanism of ACTA2-AS1 revealed that it acted as a ‘sponge’ by getting involved in a competition against miR-378a-3p binding to modify its target Wnt5a. The suppression of DDP-resistance in OC cells caused by ACTA2-AS1 downregulation was reversed by silencing miR-378a-3p. Furthermore, via inhibition of Wnt5a, miR-378a-3p alleviated DDP resistance in OC cells. These findings show that for miR-378a-3p, ACTA2-AS1 works like a sponge thus preventing it from binding to Wnt5a and boosting OC cell DDP resistance. Our research will aid the expansion of plausible therapeutic options for treating OC.

## Graphical abstract

**Figure:**
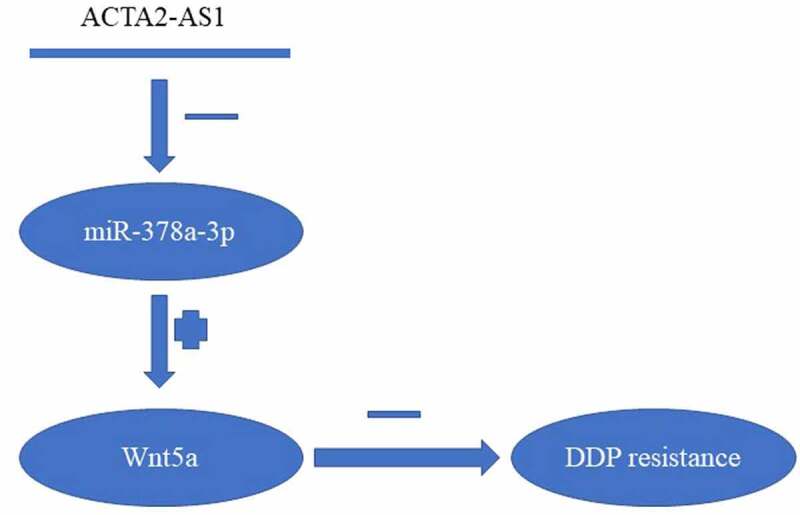

## Introduction

Ovarian cancer, a deadly disease involving the female reproductive system, is a predominant cause of female fatalities that are directly associated with cancer [1]. Cisplatin (DDP) is a first-line chemotherapy drug used to treat ovarian cancer [2]. DDP-resistance, on the other hand, is common in advanced-stage ovarian carcinoma patients and is linked to a poor prognosis [3]. As a result, it’s critical to look into the molecular fundamentals of DDP resistance within cases of ovarian carcinoma, thereby coming up with better and successful treatment options.

A form of a non-coding transcript, long non-coding RNAs (lncRNAs), were later identified as key mediators of cellular proliferation, apoptosis, and invasion in a variety of cancers [4–6]. Furthermore, lncRNAs were found to be dysregulated in a variety of malignancies, according to numerous lines of evidence. For example, the lncRNA DSCAM-AS1 undergoes interaction with Y box binding protein 1 (YBX1), thereby promoting cancer growth by activating the forkhead box A1 (FOXA1) transcription network in a positive feedback loop [7]. By disrupting DEAD box polypeptide 5 (DDX5) and reducing the MRN complex-mediated DNA restoration signals, the lncRNA SLC26A4-AS1 reduces metastasis of thyroid cancer [8]. By inhibiting miR-106b, the lncRNA PTENP1 slows the growth of cervical cancer [9]. Additionally, lncRNA can also play an important role in the chemoresistance of OC. For instance, lncRNA-PRLB confers paclitaxel resistance of ovarian cancer cells by regulating remodeling and spacing factor 1 (RSF1)/NF-kappaB signaling pathway [10]. LncRNA WDFY3-AS2 promotes cisplatin resistance and the cancer stem cell in ovarian cancer by regulating hsa-miR-139-5p/syndecan 4 (SDC4) axis [11]. It was discovered that ACTA2-AS1 acted as an oncogene in OC [12] and cervical cancer [13]. However, the function of ACTA2-AS1 in the acquisition of DDP resistance in OC has yet to be determined.

We found out that ACTA2-AS1 was substantially elevated in DDP-resistant OC patients as well as DDP-resistant OC cell lines investigated in the current investigation. We also found that ACTA2-AS1 can function as a sponge for miR-378a-3p, allowing it to bring about the upregulation of the level of Wnt5a and therefore enhance OC DDP resistance. Our findings will add to our understanding of ACTA2-AS1ʹs regulatory processes in OC DDP resistance.

## Materials and methods

### Tissue Samples and Patients

In the Department of Obstetrics and Gynecology belonging to the First People’s Hospital of Wenling, 102 samples of ovarian carcinoma were obtained from patients suffering from ovarian cancer and undergoing oophorectomies. The clinicopathological features were recorded (Table 1). Treatment-resistant patients (R, n = 57) and Treatment-sensitive patients (S, n = 45) were grouped into two categories based on the nature of their response toward first-line chemotherapy. In the light of National Comprehensive Cancer Network (NCCN) guidelines, intrinsically treatment-resistant tumors were referred to as the ones with the recurrent or persistent diseased condition during the 6 months’ duration of starting first-line DDP chemotherapy. Treatment-sensitive tumors were defined as the ones that had a 6-month full response to treatment. The First People’s Hospital of Wenling’s Ethics Committee duly passed the details of the investigation, and individual subjects provided written informed permission prior to surgery.

**Table 1.** Association between ACTA2-AS1 expression level and clinicopathological features of patients with OC

Clinicopathological features	ACTA2-AS1		P value
	Low (n = 51)	High (n = 51)	
Age, y			0.642
≤50	26	27	
>50	25	24	
Tumor size, cm			0.028
≤8	39	15	
>8	12	36	
FIGO stage			0.032
I to II	30	11	
III to IV	21	40	
Histological subtype			0.691
Serous	18	24	
Others	33	27	
Histological grade			0.754
G1 to G2	19	25	
G3	32	26	
Lymph node metastasis			0.016
Absent	35	10	
Present	16	41	

## Cell culture and transfection

Procell Life Science &Technology Co., Ltd. (Wuhan, China) provided a pair of ovarian cancer cell lines, A2780 and SKOV3, which were allowed to develop in Dulbecco’s modified Eagle’s medium (Sigma, St. Louis, MO, USA) with 10% fetal bovine serum (Sigma) and kept with 5% CO_2_.at 37°C. Proliferating cellular cultures were treated with DDP (Dalian Meilun Biotechnology Co., Ltd., Dalian, China) at an ultimate concentration of 1 M for 12 weeks resulting in drug-resistant SKOV3 and A2780 cell lines. Seeding of the cells into 6-well plates was followed by transfection with Lipofectamine 2000 (Invitrogen, Carlsbad, CA, USA) making use of the manufacturer’s outlined steps with miR-378a-3p mimic, miR-378a-3p inhibitor, negative control (NC) mimic, si-ACTA2-AS1, NC inhibitor, or NC siRNA. Drug-resistant cells were taken 48 hours after transfection for additional analysis.

## Reverse transcription-polymerase chain reaction (RT-qPCR)

TRIZOL reagent (No. S308876, Yeyuan, Shanghai, China) was employed for the extraction of total RNA, which was then transcribed into cDNA making use of an RT reaction kit (No. DD546862, Yiji, Shanghai, China). The ABI system was used to perform RT-qPCR (No. 9700, USA). internal control, glyceraldehyde-3-phosphate dehydrogenase (GAPDH), and U6 were used. Expression fold changes were ascertained by employing the 2^−ΔΔCT^ formula. The primers were showed in Table 2.

**Table 2.** The primer sequences included in this study

Name	primer sequences (5’-3’)
ACTA2-AS1	
forward	5′-GTGGTTCTGGTTTGCCTGAT-3′
reverse	5′-CTGGCCCTGTAACACCAGAT-3′
miR-382-5p	5′-CTGGACTTGGAGTCAGAAGGAA-3′
Wnt5a	
forward	5′- GTTTCGGCTACAGACCCAGA-3′
reverse	5′- CCCCAGTTCATTCACACCACA-3′
GAPDH	
forward	5′- GCACCACCAACTGCTTAGCA-3′
reverse	5′- GTCTTCTGGGTGGCAGTGATG-3′
U6	
forward	5′-CTCGCTTCGGCAGCACA-3′
reverse	5′-AACGCTTCACGAATTTGCGT-3′

## CCK-8

Following the instructions, the CCK-8 kit (No.DD546592, Yiji, Shanghai, China) was used to assess cell proliferation. In 96-well plates, cells (5 × 10^3^ cells per well) were planted. Each group received DDP at a variety of concentrations (0 μM, 5 μM, 10 μM, 20 μM, 40 μM, 80 μM). After that, the plates were allowed to incubate at 37°C for another 48 hours. CCK-8 was added to each well in a total of 10 μl, and the 96-well plates were set for incubation additionally for an hour before the absorbance (OD) at 450 nm was estimated using enzyme labeling equipment (No. DXT11008412001, Roche, USA).

## Dual-luciferase reporter gene system

StarBase predicted the ACTA2-AS1 or Wnt5a binding sites of miR-378a-3p. ACTA2-AS1 or Wnt5a binding sites were put into the pmirGLO vector to create the reporter plasmids (No. YB-0577, Ybscience, Shanghai, China). Lipofectamine 2000 was used to co-transfect MiR-378a-3p mimics and reporter plasmids into 293 T cells. The dual-luciferase Reporter Assay System (No. E1910, Promega, USA) was utilized for evaluating the activity of Firefly and Renilla luciferases after they had been cultured for 48 hours.

## RIP assay

The RIP assay was executed following the protocol detailed by the manufacturer making use of a Bersinbio Magna RIP RNA-binding protein immunoprecipitation kit (Guangzhou, China). Complete RIP lysis buffer was used to carry out the lysis of cells, which were then divided into a pair of equal parts and treated overnight at 4°C with either 5 g nonspecific anti-immunoglobulin G (IgG) antibody (Millipore) or human anti-Argonaute2 (AGO2) antibody (Millipore, Billerica, MA, USA). By using RT-qPCR, the degree of expression of the genes of interest was determined by utilizing pure RNA.

## Wnt5a Elisa

An ELISA kit (R&D Systems) was used to measure Wnt5a levels in various culture mediums according to the manufacturer’s recommendations.

## Statistical analyses

This project’s experiments were carried out three times. The findings were presented as mean SD, and statistical analysis was conducted by utilizing GraphPad Prism 7 and a t-test or one-way ANOVA. P < 0.05 was considered statistically significant.

## Results

In DDP-resistant cells and tissues, ACTA2-AS1 was increased, while a substantial downregulation in miR-378a-3p was noticed. In cells manifesting DDP-resistance, knocking down ACTA2-AS1 boosted the expression of miR-378a-3p. Further research into the mechanism of ACTA2-AS1 revealed that it acted as a ‘sponge’ by getting involved in a competition against miR-378a-3p binding to modify its target Wnt5a. The suppression of DDP-resistance in OC cells caused by ACTA2-AS1 downregulation was reversed by silencing miR-378a-3p. Furthermore, via inhibition of Wnt5a, miR-378a-3p alleviated DDP resistance in OC cells.

## ACTA2-AS1 is increased in DDP‑resistant OC cell lines and tissues

**Figure 1.**
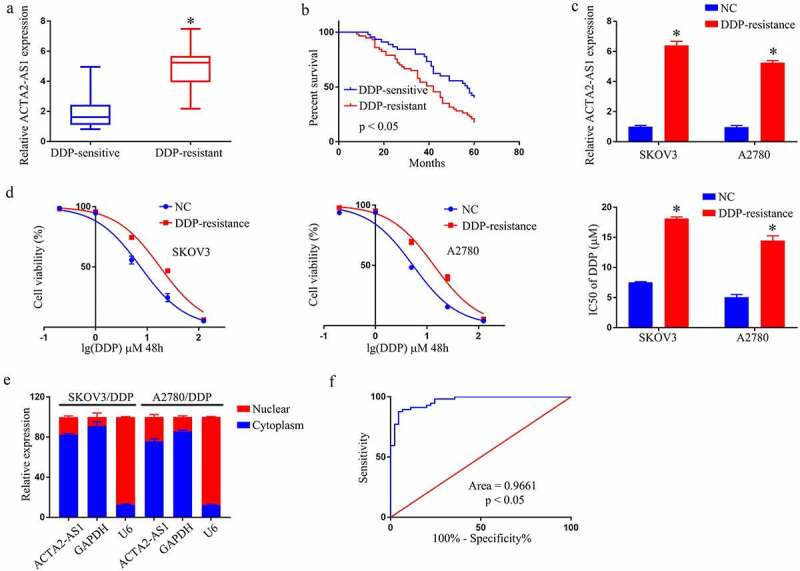
ACTA2-AS1 is up-regulated in DDP‑resistant OC cell lines and tissues (a) ACTA2-AS1 expression levels in tissues from OC patients (DDP-sensitive and DDP-resistant). (b) Kaplan-Meier survival curves for patients with DDP-sensitive and DDP-resistant. (c) Expression of ACTA2-AS1 in DDP-resistant cell lines. (d) CCK-8 assay of the viability and IC50 value of OC/DDP cells. (e) ACTA2-AS1 from the nuclear and cytoplasmic fractions of OC/DDP cells. (f) ROC curves of ACTA2-AS1. *p < 0.05.

Figure 1a shows a rising trend in ACTA2-AS1 levels from OC tissues that are DDP-sensitive to the resistant ones, with DDP-resistant patients having a considerably shorter overall life (Figure 1b). Experiments at the cellular level were then carried out. The expression of ACTA2-AS1 was significantly higher in SKOV3/DDP and A2780/DDP cells (Figure 1c). Furthermore, as evident from Figure 1d, DDP resistance was dramatically improved in A2780/DDP and SKOV3/DDP cells. The findings of the RT-qPCR showed that ACTA2-AS1 was enriched in the cytoplasm (Figure 1e). ACTA2-AS1ʹs receiver operating characteristic (ROC) curves suggested that it could be a worthy marker for predicting OC patients’ resistance toward DDP. (figure 1f).

## ACTA2-AS1 knockdown blocks DDP resistance of OC/DDP cells in vitro

**Figure 2.**
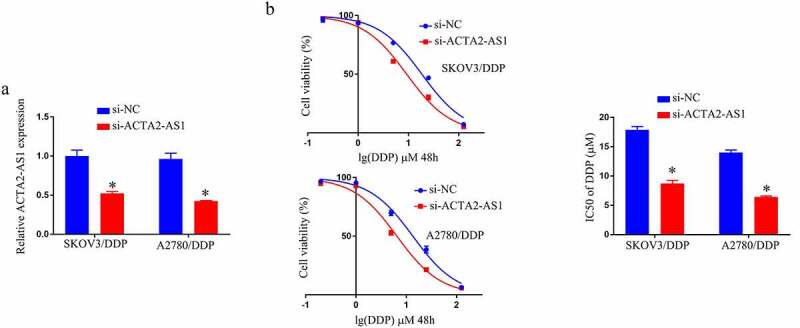
ACTA2-AS1 knockdown inhibits DDP resistance of OC/DDP cells in vitro. (a) RT-qPCR analysis of ACTA2-AS1 expression in OC/DDP cells. (b) CCK-8 assay of the viability and determination of IC_50_ value of OC/DDP cells following exposure to DDP as a series dose. *p < 0.05.

We knocked down ACTA2-AS1 in A2780/DDP and SKOV3/DDP cells, and validated the degree of expression of ACTA2-AS1 using RT-qPCR in SKOV3/DDP as well as A2780/DDP cells, as demonstrated in Figure 2a. In comparison to the si-NC group, the IC50 value of DDP was lower in the si-ACTA2-AS1 group (Figure 2b). In vitro, knocking down ACTA2-AS1 decreases DDP resistance in OC cells.

## Silencing ACTA2-AS1 sensitizes OC cells to DDP by targeting miR-378a-3p

**Figure 3.**
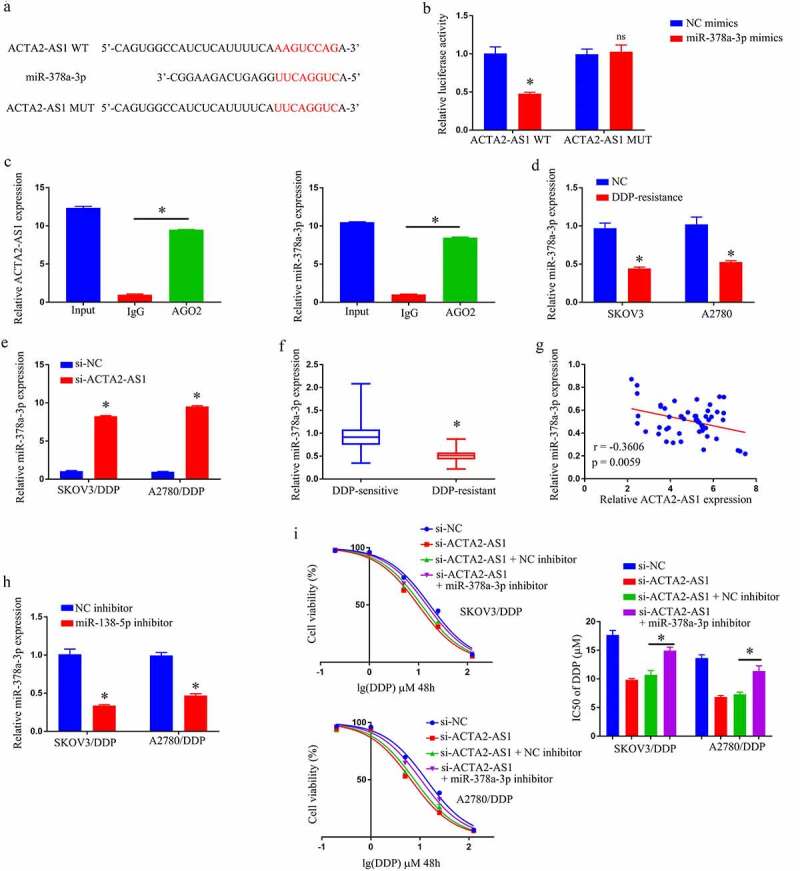
Silencing ACTA2-AS1 sensitizes OC cells to DDP by targeting miR-378a-3p. (a) For miR-378a-3p, ACTA2-AS1 is thought to act as a molecular sponge. (b) Luciferase assays in HEK293T cells co-transfected with mutant or wild-type ACTA2-AS1 and miR-378a-3p. (c) In HEK293T cells, anti-AGO2 RIP was conducted, followed by RT-qPCR for the detection of the expression of ACTA2-AS1 or miR-378a-3p linked to AGO2. (d) Expression of miR-378a-3p in DDP-resistant cell lines. (e) The effect of ACTA2-AS1 knockdown on the expression of miR-378a-3p in OC/DDP cells was investigated using RT-qPCR. (f) MiR-378a-3p expression levels in tissues from individuals suffering from OC (DDP-sensitive and DDP-resistant). (g) A negative correlation between ACTA2-AS1 expression and miR-378a-3p expression in DDP-resistant OC tissues. (h) RT-qPCR analysis of miR-378a-3p in OC/DDP cells transfected with NC inhibitor or miR-378a-3p inhibitor. (i) CCK-8 assay of the viability and determination of IC_50_ value of OC/DDP cells upon exposure to DDP as a series dose. *p < 0.05, ^ns^p > 0.05.

Prediction of the target genes of ACTA2-AS1 was made using a bioinformatics database to be miR-378a-3p (Figure 3a). miR-378a-3p up-regulation was observed to lower the luciferase activity of the ACTA2-AS1 WT reporter, nevertheless produced no impact on the ACTA2-AS1 MUT reporter, in the light of the results of a dual-luciferase reporter gene system (Figure 3b). The RIP experiment also clearly demonstrated that miR-378a-3p was an ACTA2-AS1ʹs target genes (Figure 3c). Furthermore, RT-qPCR disclosed that miR-378a-3p expression was much lower in OC/DDP cells (Figure 3d), and downregulation of ACTA2-AS1 significantly enhanced the degree of expression of miR-378a-3p in OC/DDP cells as illustrated in Figure 3e. Furthermore, when compared to DDP-sensitive OC tissues, the expression level of miR-378a-3p in DDP-resistant OC tissues was much lower (figure 3f). In OC tissues that manifested DDP-resistance, a negative connection was found to exist among ACTA2-AS1 levels and miR-378a-3p (Figure 3g). Furthermore, as evident from Figure 3h, the relative expression of miR-378a-3p in OC/DDP cells was considerably reduced upon treatment with a miR-378a-3p inhibitor. The CCK-8 experiment revealed that si-ACTA2-AS1 lowered DDP’s IC50 value, which was considerably reversed in OC/DDP cells by a miR-138-5p inhibitor (Figure 3i). By targeting miR-378a-3p, we were able to confirm that silencing ACTA2-AS1 sensitizes OC cells to DDP.

## MiR-378a-3p diminishes DDP resistance in GC cells by targeting Wnt5a

**Figure 4.**
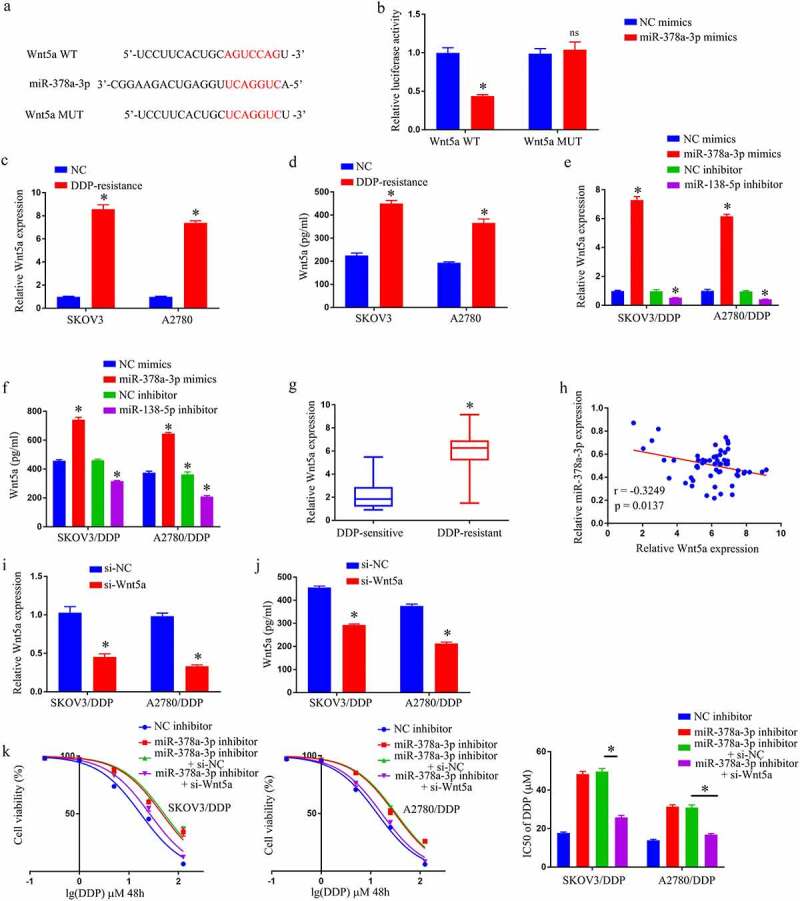
By targeting Wnt5a, MiR-378a-3p reduces DDP resistance in GC cells. (a) Wnt5a was predicted to function as a molecular sponge for miR-378a-3p. (b) Luciferase assays in HEK293T cells co-transfected with mutant or wild-type Wnt5a and miR-378a-3p. Expression of Wnt5a in DDP-resistant cell by RT-qPCR (c) and Elisa assay (d). Detection of expression of Wnt5a in OC/DDP cells that have been transfected with NC inhibitor, miR-378a-3p mimics, miR-378a-3p inhibitor, or NC mimics using RT-qPCR (e) and Elisa assay (f). (g) Wnt5a expression levels in tissues from OC patients (DDP-resistant and DDP-sensitive). (h) In DDP-resistant OC tissues, there is a negative connection between Wnt5a expression and miR-378a-3p expression. Expression detection of Wnt5a in OC/DDP cells transfected with si-NC or si-Wnt5a using RT-qPCR (i) and Elisa assay (j). (k) CCK-8 assay of the viability and determination of IC_50_ value of OC/DDP cells after exposure to DDP.as a series dose *p < 0.05, ^ns^p > 0.05.

TargetScan predicted that miR-378a-3p will target Wnt5a (Figure 4a). Reporters with WT miR-378a-3p bind-sites at the Wnt5a 3ʹUTR displayed reduced luciferase activity than constructions with bind-site mutations in 293 T co-transfected using miR-378a-3p mimics (Figure 4b). Wnt5a was observed to be substantially increased in OC/DDP cells using RT-qPCR and Elisa (Figure 4c –d). Transfected miR-378a-3p inhibitor considerably enhanced Wnt5a expression in OC/DDP cells, whereas transfected miR-378a-3p mimic substantially lowered Wnt5a expression in OC/DDP cells (Figure 4e –f). Wnt5a was also shown to be significantly elevated in DDP-resistant OC tissues (Figure 4g). Furthermore, in OC tissues, a negative connection existed among miR-378a-3p levels and Wnt5a (Figure 4h). The miR-378a-3p/Wnt5a axis was then tested to see if it influenced DDP resistance in OC/DDP cells. It was demonstrated that si-Wnt5a transfection drastically reduced Wnt5a expression in OC/DDP cells when compared to their counterparts (Figure 4i –j). After that, a rescue experiment was carried out, and the findings revealed that miR-378a-3p inhibitor caused a notable enhancement in the IC50 value of DDP in OC/DDP cells, whereas si-Wnt5a completely eliminated this effect (Figure 4k). By targeting Wnt5a, miR-378a-3p alleviates DDP resistance in GC cells.

## ACTA2-AS1 positively regulates Wnt5a through sponging miR-378a-3p

**Figure 5.**
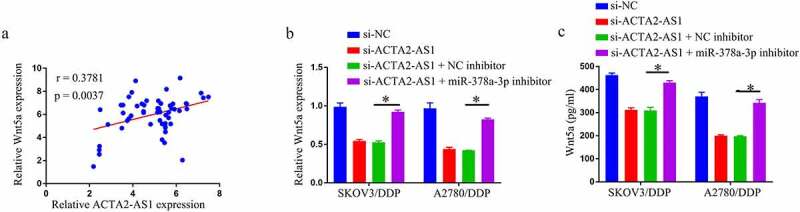
ACTA2-AS1 positively regulates Wnt5a through sponging miR-378a-3p. (a) Correlation analysis between ACTA2-AS1 and Wnt5a expression in DDP-resistant OC tissues. Expression detection of Wnt5a in OC/DDP cells using RT-qPCR (b) and Elisa assay (c). *p < 0.05.

Given that miR-378a-3p targeted Wnt5a and that ACTA2-AS1 was a miR-378a-3p sponge, it was investigated if ACTA2-AS1 regulated Wnt5a by competing for miR-378a-3p binding. First, in OC tissues, there was a positive connection between ACTA2-AS1 and Wnt5a levels (Figure 5a). Then, using qRT-PCR and Elisa analysis, we discovered that miR-378a-3p suppression dramatically reversed the si-ACTA2-AS1-triggered reduction of Wnt5a level in OC/DDP cells (Figure 5b –c). Overall, ACTA2-AS1 may influence Wnt5a expression indirectly via miR-378a-3p.

## Discussion

DDP resistance is a critical problem encountered when treating OC, that must be addressed immediately. The link between DDP sensitivity and ACTA2-AS1 expression level in patients suffering from OC was investigated in this study. The findings demonstrated that ACTA2-AS1 functioned as a promoter of DDP resistance in OC through the miR-378a-3p/Wnt5a axis after loss-of-function tests in vitro. ACTA2-AS1 was found to be increased in DDP-resistant OC patients and OC cell lines in this study. ACTA2-AS1 was also discovered to be a regulator of OC progression. For example, in the development of ovarian cancer, the mechanism underpinning the control of lncRNA ACTA2-AS1 on chemokine ligand 2 (CXCL2) by absorbing miRNA-532-5p as ceRNA [12]. Increased research has revealed that lncRNAs have an indispensable role in treating different malignancies, including gastric cancer [14,15], breast cancer [16,17], and pancreatic cancer [18,19]. Furthermore, our findings contribute to the growing body of evidence that ACTA2-AS1 might have a crucial function in developing DDP resistance in OC patients.

LncRNAs had long been thought to be the product of gene transcription mistakes, but new data suggests they are more likely to be regulators of specialized physiological tasks. LncRNA has several activities, including regulating transcription and splicing, functioning as miRNA sponges, and interacting with various proteins [20–22]. The role of lncRNA as a miRNA sponge has gotten a lot of attention recently. By sequestering various miRNAs, we discovered that lncRNA could improve cisplatin resistance in OC. NEAT1 knockdown, for example, reduces cisplatin resistance in OC via control of the miR-770-5p/PARP1 axis [23]. MALAT1 modulates the miR-1271-5p/ E2F transcription factor 5 (E2F5) axis, which controls OC cell growth and cisplatin resistance [24].

ACTA2-AS1 was discovered to cause the formation of DDP resistance by targeting miR-378a-3p, which caused a considerable decline in Wnt5a expression in the current investigation. By targeting mitogen-activated protein kinase 1 (MAPK1)/growth factor receptor bound protein 2 (GRB2), MiR-378a-3p makes ovarian cancer cells more sensitive to cisplatin [25]. Moreover, miR-378a-3p influences chemotherapy sensitivity in a variety of cancers [26,27]. Wnt5a also activates the Wnt5a/protein kinase C (PKC) signaling pathway, which promotes lung cancer stem cell characteristics and resistance to cisplatin [28]. Wnt5a also boosts cisplatin resistance in OC [29]. Our results were identical to those mentioned previously. DDP-resistant OC tissues and cell lines have greater levels of Wnt5a. In addition, we discovered that knocking down ACTA2-AS1 or overexpressing miR-378a-3p in OC/DDP cells led to a substantial decline in Wnt5a. The findings show that an ACTA2-AS1/miR-1252/forkhead box R2 (FOXR2) axis exists in OC and is involved in cisplatin resistance modulation. The mechanism by which Wnt5a modulates DDP resistance, as well as the clinical significance of the ACTA2-AS1/miR-378a-3p/Wnt5a axis, are still unknown.

## Conclusions

**Figure 6.**
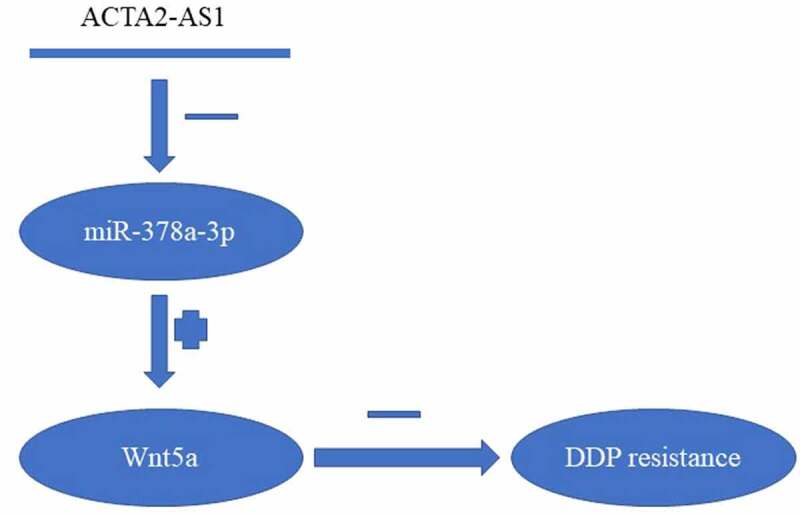
Summary of ACTA2-AS1/miR-378a-3p/Wnt5a axis.

We found out that the overexpression of ACTA2-AS1 in DDP-resistant OC functioned as a biomarker for poor prognosis. Mechanical knockdown of ACTA2-AS1 in OC cell lines could overcome DDP resistance by targeting the miR-378a-3p/Wnt5a axis (Figure 6). Furthermore, our research could potentially contribute toward the advancement of therapeutic techniques for the treatment of OC.
